# Circulating immune-complexes of IgG/IgM bound to B2-glycoprotein-I associated with complement consumption and thrombocytopenia in antiphospholipid syndrome

**DOI:** 10.3389/fimmu.2022.957201

**Published:** 2022-09-12

**Authors:** Laura Naranjo, Ljudmila Stojanovich, Aleksandra Djokovic, Laura Andreoli, Angela Tincani, Maria Maślińska, Savino Sciascia, Maria Infantino, Sara Garcinuño, Kinga Kostyra-Grabczak, Mariangela Manfredi, Francesca Regola, Natasa Stanisavljevic, Milomir Milanovic, Jovica Saponjski, Dario Roccatello, Irene Cecchi, Massimo Radin, Maurizio Benucci, Daniel Pleguezuelo, Manuel Serrano, Yehuda Shoenfeld, Antonio Serrano

**Affiliations:** ^1^ Immunology Department, Hospital Universitario 12 de Octubre, Madrid, Spain; ^2^ Internal Medicine, University Hospital Center Bezanijska Kosa, Belgrade, Serbia; ^3^ Cardiology Department, University Hospital Center Bezanijska Kosa, Belgrade, Serbia; ^4^ School of Medicine , University of Belgrade, Belgrade, Serbia; ^5^ Unit of Rheumatology and Clinical Immunology, ASST Spedali Civili, Brescia, Italy; ^6^ Department of Clinical and Experimental Sciences, University of Brescia, Brescia, Italy; ^7^ Early Arthritis Clinic, National Institute of Geriatrics, Rheumatology and Rehabilitation, Warsaw, Poland; ^8^ Nephrology and Dialysis Unit (ERK-net Member), Center of Research of Immunopathology and Rare Diseases, Coordinating Center of the Network for Rare Diseases of Piedmont and Aosta Valley, San Giovanni Bosco Hospital, Torino, Italy; ^9^ Immunology and Allergy Laboratory, San Giovanni di Dio Hospital, Florence, Italy; ^10^ Internal Medicine Department, Clinic for Infectious and Tropical Diseases, Military Medical Academy, Belgrade, Serbia; ^11^ Cardiology Department, University Clinical Center of Serbia, Belgrade, Serbia; ^12^ Rheumatology Unit, San Giovanni di Dio Hospital, Florence, Italy; ^13^ Ariel University, Ariel, Israel; ^14^ Zabludowicz Center for Autoimmune Diseases, Sheba Medical Center, Tel-Hashomer, Israel

**Keywords:** circulating immune-complexes, antiphospholipid syndrome, complement factors, platelets, thrombocytopenia

## Abstract

**Background:**

Antiphospholipid syndrome (APS) is a multisystemic autoimmune disorder characterized by thrombotic events and/or gestational morbidity in patients with antiphospholipid antibodies (aPL). In a previous single center study, APS-related clinical manifestations that were not included in the classification criteria (livedo reticularis, thrombocytopenia, leukopenia) were associated with the presence of circulating immune-complexes (CIC) formed by beta-2-glycoprotein-I (B2GP1) and anti-B2GP1 antibodies (B2-CIC). We have performed a multicenter study on APS features associated with the presence of B2-CIC.

**Methods:**

A multicenter, cross-sectional and observational study was conducted on 303 patients recruited from six European hospitals who fulfilled APS classification criteria: 165 patients had primary APS and 138 APS associated with other systemic autoimmune diseases (mainly systemic lupus erythematosus, N=112). Prevalence of B2-CIC (IgG/IgM isotypes) and its association with clinical manifestations and biomarkers related to the disease activity were evaluated.

**Results:**

B2-CIC prevalence in APS patients was 39.3%. B2-CIC-positive patients with thrombotic APS presented a higher incidence of thrombocytopenia (OR: 2.32, p=0.007), heart valve thickening and dysfunction (OR: 9.06, p=0.015) and triple aPL positivity (OR: 1.83, p=0.027), as well as lower levels of C3, C4 and platelets (p-values: <0.001, <0.001 and 0.001) compared to B2-CIC-negative patients. B2-CIC of IgM isotype were significantly more prevalent in gestational than thrombotic APS.

**Conclusions:**

Patients with thrombotic events and positive for B2-CIC had lower platelet count and complement levels than those who were negative, suggesting a greater degree of platelet activation.

## Introduction

Antiphospholipid syndrome (APS) is defined as a multisystemic autoimmune disorder characterized by the occurrence of thrombotic events and/or gestational morbidity in presence of persistent antiphospholipid antibodies (aPL). Several clinical forms of the syndrome can be identified: 1) APS associated with other systemic autoimmune diseases (SAD-APS) such as systemic lupus erythematosus (SLE) or rheumatoid arthritis (RA), 2) APS without other associated diseases (primary APS) and 3) catastrophic APS (patients with thrombosis in multiple sites of the vasculature resulting in multiorgan failure with a high mortality rate) (1, 2). Current APS classification criteria require the presence of at least one clinical and one laboratory criterion (3). Clinical criteria include small vessel, arterial or venous thrombosis in any organ or tissue and/or gestational morbidity. Laboratory criteria consist of the presence of some of the following aPL: lupus anticoagulant (LA), anti-cardiolipin (aCL) or anti-β2-glycoprotein-I (aB2GP1) antibodies of IgM or IgG isotype. Positivity must be confirmed at least 12 weeks after the first measurement.

In addition to the clinical manifestations included in the classification criteria, there are other non-criteria clinical features strongly associated with APS such as heart valve disease (4), neurological manifestations (5), livedo reticularis (6) and thrombocytopenia (7). Similarly, other non-criteria autoantibodies have been associated with APS, highlighting the presence of IgA aB2GP1 (8), anti-domain-I of B2GP1 antibodies (9) and IgG/IgM anti-phosphatidylserin/prothrombin antibodies (aPS/PT) (10). Although these antibodies and clinical manifestations are associated with APS, they were not included in the last 2006 criteria update due to the limited evidence at that time (11).

Persistent presence of aPL in asymptomatic carriers implies a paradoxical situation. Although both the antibodies and the main antigen to which they bind (B2GP1) coexist permanently in circulation, thrombotic events only occur occasionally in aPL carriers (risk of developing a thrombotic event in patients with aPL is 3% per year) (12, 13). To explain this situation, the two-hit hypothesis was proposed: antibodies induce a pro-thrombotic state that is necessary but insufficient to trigger thrombotic events and clotting takes place only in the presence of another facilitating condition (second hit) (14). Vascular lesions, infections, inflammatory factors or other procoagulant conditions (such as surgery and immobility) are factors that have been described as triggers of APS events (15).

It has been described that B2GP1 has several conformations. The open (J-shaped) and circular (closed or O-shaped) forms are the most studied conformations. B2GP1 circulates in blood in >99% in a circular conformation. During certain conditions, such as a systemic inflammation or a strong activation of the innate immune system, it is known that the molecule “opens” in a J-shape conformation, exposing cryptic epitopes (such as in domain I) which are well-known targets of aPL. Thus, the consequences of a second hit at molecular level would be the activation of the B2GP1 with a conformational change, involving the exposure of previously cryptic epitopes (16, 17). In addition to the pathogenic aPL directed against domain I of B2GP1, there are also other thrombosis-associated aPL that recognize epitopes in domains 3 and 4 of the protein (18, 19), whose pathogenicity has been demonstrated by animal models (20). Recently, using hydrogen/deuterium exchange techniques, it has been described that these epitopes in domains 3 and 4 are also located in hidden areas in the closed conformation of B2GP1, which can only be exposed after the activation of the protein and its conversion into the open form (21).

Several authors have emphasized the need for additional biomarkers to improve the assessment of disease activity in APS patients and to identify patients at risk of suffering such events (22, 23). The presence of immune-complexes formed by IgA aB2GP1 bound to B2GP1 (B2A-CIC) has been described in patients with thrombotic manifestations of APS (24, 25). Their presence during the thrombotic event was a risk factor for the development of acute thrombosis (OR 22.7, 95% CI: 5.06-101.57) (24). Besides, the presence of B2A-CIC was evaluated in patients undergoing a renal transplantation. Pre-transplant presence of B2A-CIC was an independent risk factor for graft thrombosis in the first 6 months post-transplant (HR: 14.75, 95% CI: 9.11-23.89) (26). In addition, in the case of patients undergoing cardiac transplantation, post-transplant incidence of thrombosis was significantly higher in B2A-CIC positive patients (OR: 6.42, 95% CI: 2.1-19.63) (27).

Additionally, the presence of immune-complexes constituted by IgG or IgM aB2GP1 bound to B2GP1 (B2G-CIC and B2M-CIC, respectively) was described in a Serbian cohort of APS patients (28). The prevalence of these immune-complexes overall (B2-CIC) was 19.3% and they were associated with an increased incidence of livedo reticularis, ocular dryness, thrombocytopenia and leukopenia as well as with a decrease in the levels of complement factors C3 and C4. However, this study was performed in a single center with a limited number of patients.

In agreement with these results, Taatjes et al. visualized the formation of immune-complexes using sera from APS patients by high resolution microscopy-based imaging techniques (29). Prior to these studies, a small number of reports were published that suggested the presence of B2-CIC in patients with SLE and APS by heparin affinity chromatography (30, 31). However, the complexity of this technology has hampered its use in the daily clinical practice.

Our study has aimed: 1) to develop an easy to use and reproducible method to detect the presence of B2G and B2M-CIC in patients with suspected APS; 2) to determine the prevalence of B2G and B2M-CIC in a multicenter cohort of APS patients; and 3) to analyze the association of B2G/B2M-CIC presence with signs that indicate pathogenic activity of aPL such as the presence of clinical manifestations and laboratory parameters in order to identify patients having a higher thrombotic risk.

## Materials and methods

### Study design

A cross-sectional, retrospective and multicenter study was performed to determine the prevalence of B2G and B2M-CIC in an APS patient cohort recruited from six European centers, and their association with APS-related clinical and laboratory parameters.

### Patients

A total of 303 patients classified as APS were consecutively recruited from six European hospitals during the year 2019:

1) University Hospital Center Bezanijska Kosa, Internal Medicine and Cardiology Units (Belgrade, Serbia) (N=119).

2) National Institute of Geriatrics, Rheumatology and Rehabilitation, Rheumatology Unit (Warsaw, Poland) (N=36)

3) ASST Spedali Civili di Brescia, Rheumatology and Clinical Immunology Unit (Brescia, Italy) (N=38)

4) San Giovanni di Dio Hospital, Rheumatology Unit (Florence, Italy) (N=15)

5) San Giovanni Bosco Hospital, Nephrology and Dialysis Unit (Torino, Italy) (N=66)

6) Hospital Universitario 12 de Octubre, Immunology Department (Madrid, Spain) (N=29).

*Inclusion criteria*. All patients were examined at their origin center by physicians specialized in Internal Medicine, Rheumatology, Immunology, Neurology, Cardiology or Hematology. Those patients with an APS diagnosis who met the revised classification criteria (both clinical and laboratory criteria) were enrolled in the study (3). Patients were differentiated according to the type of APS event they suffered: 1) isolated thrombotic APS, 2) isolated gestational morbidity, 3) mixed APS (patients who have suffered both thrombotic and gestational manifestations), 4) total thrombotic APS (patients with some thrombotic event, that is, isolated thrombotic and mixed APS) and 5) total gestational morbidity (patients with isolated gestational morbidity and mixed APS).

All SLE-diagnosed patients met the American College of Rheumatology classification criteria (32, 33). Disease activity was assessed at the time of study enrolment using the Systemic Lupus Erythematosus Disease Activity Index (SLEDAI) and only patients with a stable disease were included.

Laboratory and clinical data were obtained from the patient’s clinical records at their respective center. The presence of APS clinical criteria, systemic autoimmune diseases, comorbidities (arterial hypertension, diabetes, smoking, dyslipidemia), treatments, laboratory parameters and a series of clinical manifestations associated with APS were included in the data collection form sent to all participating centers.

*Exclusion criteria* included chronic or acute infections, marked renal/hepatic impairment, presence of present/treated malignancy or patients younger than 18 years.

### Laboratory measurements

LA, aCL and aB2GP1 antibodies (of IgG and IgM isotypes) were evaluated at the corresponding centers where patients were recruited, using serum (aCL and aB2GP1 antibodies) or plasma samples (LA) at the same time prior to inclusion or in the blood analysis at enrolment in the study (described in **Supplementary Methods**). The cutoff used for each measurement calculated based on the 99th percentile of a healthy population or following the manufacturer’s guidelines was specific to the local laboratory where quantification was made.

LA evaluation was performed by coagulation assays according to the recommendations of the International Society on Thrombosis and Hemostasis (ISHT) (34). It was based on the prolongation of clotting time by two screening tests (activated partial thromboplastin time or dilute Russell viper venom time), the failure to correct this time by mixing the patient’s sample with a normal donor and the correction of clotting time after addition of phospholipids excess. LA tests were performed while patients were not receiving anticoagulant therapy.

After aPL evaluation, samples were stored at -20°C and sent to the Autoimmunity Laboratory at Hospital Universitario 12 de Octubre (Madrid) for the quantification of B2G and B2M-CIC.

Complement factors C3 and C4 were determined by nephelometry using the Beckman Coulter IMMAGE Immunochemistry System (Beckman Coulter Inc. Pasadena, CA, USA). The normal range for C3 levels was 88-225 mg/dl and for C4 levels 12-75 mg/dl. Quantitative detection of human C3a and C5a was performed in 51 patients using the commercial kits Human C3a ELISA and Human C5a ELISA (Invitrogen, ThermoFisher Scientific, Waltham, USA).

### Quantification of B2G and B2M-CIC

B2G and B2M-CIC measurement was performed by ELISA. In order to achieve a stable calibration system for transforming optical densities (OD) values into Units/ml, recombinant B2-CIC molecules were generated by transduction of human embryonic kidney cells (HEK293) with lentiviral vectors. The molecules consisted of chimeric proteins containing the Fc region of the corresponding immunoglobulin isotype fused to B2GP1. For the IgG-fused B2GP1 calibrator, a cDNA fragment of human IgG1 that included the hinge region, the constant domains 2 and 3 and the stop codon was incorporated (hinge and Fc regions). For the B2GP1-IgM chimeric protein, the same procedure was performed. In this case, the cDNA encoding the constant domains 2, 3 and 4 of the mu-heavy chain was fused (IgM does not have a hinged region). The final products were tested to ensure that the immunogenicity of each individual proteins (B2GP1 and immunoglobulins heavy chain) was preserved. DNA encoding the calibrator was incorporated into lentiviral vectors, packaged and transduced into HEK293T cells. The proteins were purified from the culture supernatant of recombinant cell lines with stable expression. The procedures for B2G and B2M-CIC production are currently under patent (ES2727261).

Briefly, 96-well Nunc MaxiSorp™ plates (Invitrogen, ThermoFisher Scientific, Waltham, USA) were coated overnight at 4°C with a mouse anti-human B2GP1 monoclonal antibody (ApoH mAb H219, MabTech, Nacka Strand, Sweden) at 2 μg/ml in PBS pH 7.4 for the quantification of B2G and B2M-CIC. Plates were washed 3 times (with PBS-0.1% Tween20), blocked with PBS containing 2% skim milk and 2% polyvinylpyrrolidone (Sigma-Aldrich, Merck KGAA, Darmstadt, Germany) for 1 hour at room temperature (RT) and subsequently washed. Serum samples were diluted at 1:200 in a diluent solution (PBS-0.1% Tween20-0.5% skim milk-0.5% PVP) and serial dilutions were performed for B2G and B2M-CIC calibrators. Diluted samples/blank/calibration curves were dispensed (100 μl) and incubated for 1.5 hours at RT. Plates were washed and incubated for 30 minutes at RT with an anti-human IgG (Anti-human IgG (Fc specific)-Peroxidase antibody, Sigma-Aldrich) or an anti-human IgM antibody (Anti-human IgM (µ-chain specific)-Peroxidase antibody, Sigma-Aldrich) for the detection of B2G and B2M-CIC, respectively. The reaction was revealed by incubation with TMB for 30 minutes (Invitrogen, ThermoFisher Scientific, Waltham, USA) and the addition of 0.344 M sulfuric acid. OD values were measured at 450 nm.

In each assay, sera from three B2-CIC-positive patients (with low, medium and high values) were incorporated as positive controls, as well as sera from three negative controls. The concentration of B2G and B2M-CIC (U/ml) of each patient was obtained by interpolating the optical densities values with the corresponding calibration curve. The suitability of calibration curves was verified by the concordance of the values obtained for the sera with known B2-CIC levels. B2G-CIC and B2M-CIC levels higher than 21 U/ml were considered positive, based on the 99th percentile of a healthy population (213 blood donors from Spain, Italy and Poland). The procedure was performed on the Triturus^®^ Analyzer (Diagnostics Grifols, S.A. Barcelona, Spain).

### Evaluation of antiphospholipid immune-complexes by classical methodologies

To confirm that B2-CIC-positive patients detected with the described capture ELISA were also positive by other traditional immune-complexes detection methodologies, six patients with thrombotic APS and positive for B2G-CIC, as well as two negative controls, were studied using Polyethylene Glycol 6000 (PEG-6000) to precipitate immune-complexes, if present.

Immune-complexes were isolated from serum samples by a modification of the Digeon method (35). Briefly, 100 µl of serum were added to 3.9 ml of 3.5% (w/v) of PEG-6000 (ThermoFisher Scientific) in 0.1 M borate buffer pH 8.4. It was incubated for 24 hours at 4°C and the precipitate was pelleted by centrifugation for 15 minutes at 2500 g. The pellet was redissolved in 1 ml of 0.015M PBS, pH 7.4 for 5 minutes at 37°C by gentle shaking. The redissolved immune-complexes were tested by the B2-CIC detection ELISA. Three Nunc MaxiSorp™ plates (Invitrogen, ThermoFisher Scientific) were coated overnight at 4°C with an anti-human B2GP1 monoclonal antibody (ApoH mAb H219, MabTech, Nacka Strand, Sweden) at 2 μg/ml in PBS pH 7.4. Plates were washed 3 times (with PBS-0.1% Tween20), blocked with PBS containing 2% skimmed milk and 2% polyvinylpyrrolidone (Sigma-Aldrich, Merck KGAA, Darmstadt, Germany) for 1 hour at RT and subsequently washed. Polyethylene glycol precipitates from patients and controls were added to each of the three plates and incubated for 1 hour at RT. Plates were washed and revealed using the following secondary antibodies:

Plate 1. Anti-human IgG (Fc-specific)-Peroxidase-antibody (Sigma-Aldrich).

Plate 2. Anti-human C1q antibody conjugated to peroxidase (BioRad, Hercules, CA, USA).

Plate 3. Anti-human ApoH/B2GP1-HRP conjugated (Novus Biologicals, Briarwood, CO, USA).

All plates were incubated for 45 minutes at RT and washed three times. The colorimetric reaction was achieved by incubation with TMB for 30 minutes (Invitrogen, Thermofisher) and the addition of 0.344 M sulfuric acid. OD values were measured at 450 nm.

### Immune-complexes stability

To demonstrate that B2-CIC are stable and do not form *de novo* during the detection assay, total IgG from the previous six B2G-CIC-positive patients and two negative controls were purified using Dynabeads Protein G (Invitrogen, ThermoFisher Scientific), following the manufacturer’s recommendations. Eluted immunoglobulins were adjusted to a concentration of 5 mg/dl in PBS pH 7.4, which is equivalent to a 1:200 serum dilution (the same dilution used in the assay to quantify B2-CIC).

Nunc MaxiSorp™ plates (Invitrogen, ThermoFisher Scientific) were coated overnight at 4°C with the anti-human B2GP1 monoclonal antibody (ApoH mAb H219, MabTech, Nacka Strand, Sweden) at 2 μg/ml in PBS pH 7.4. Plates were washed three times and blocked. After subsequent washes, 100 µl of purified human B2GP1 (MabTech, Nacka Strand, Sweden) at 10 ng/ml in PBS were added and incubated for 1 hour at RT. For each patient/control, three conditions were used: 1) problem well, in which 100 µl of the purified IgG diluted equivalently to a 1:200 serum dilution were added; 2) positive control and 3) negative control wells, to which 100 µl of PBS were added. After the incubation for 1 hour at RT, plates were washed. In the problem and negative control wells, 100 µl of an anti-human IgG (Fc-specific)-Peroxidase antibody (Sigma-Aldrich) were added. To the positive control wells, 100 µl of an anti-human ApoH/B2GP1-HRP conjugated (Novus Biologicals, Briarwood, CO, USA) were dispensed. Plates were incubated for 45 minutes and washed three times. The reaction was revealed by incubation for 30 minutes with TMB (Invitrogen, ThermoFisher Scientific) and the addition of 0.344 M sulfuric acid. OD values were measured at 450 nm.

In addition, the capacity of purified IgG from patients to recognize B2GP1 in the open conformation was assessed by a commercial ELISA (Orgentec Diagnostika GmbH, Mainz, Germany). IgG concentration was adjusted to be treated as a 1:100 dilution of serum (manufacturer’s recommended dilution).

### Definitions

*Thrombotic event*: small vessel, arterial or venous thrombosis in any organ or tissue. The diagnosis must be confirmed by validated objective criteria, such as imaging techniques (3).

*Gestational morbidity*: death of a morphologically normal fetus, premature delivery or spontaneous abortions defined according to the international classification criteria for APS (3).

*Triple aPL positivity:* presence of the three laboratory markers associated with APS in the same patient (LA, aCL and aB2GP1 antibodies) (3).

*Quadruple aPL positivity*: patients with triple aPL positive who are also positive for B2-CIC.

*Arterial hypertension:* systolic blood pressure >140 mmHg or diastolic blood pressure >90 mmHg, recorded on different days during evaluations (with evidence of at least 2 readings) or use of antihypertensive medication.

*Diabetes mellitus*: hyperglycemia resulting from defects in insulin secretion, insulin action, or both (at least 7% of glycated hemoglobin or use of medication).

*Dyslipidemia*: serum cholesterol concentrations >220 mg/dL, LDL >130 mg/dL, triglycerides >150 mg/dL or use of medication in patients with a history of dyslipidemia.

*Smoking*: was considered in active or former smokers (individuals who quit smoking less than 6 months ago).

*Thrombocytopenia*: number of platelets <150,000 per microliter of blood.

*Systemic lupus erythematosus* (SLE): systemic autoimmune disease whose initial diagnosis was based on the 1997 ACR classification criteria (at least 4 of the 11 criteria were present) (32, 33). These patients would also meet the 2018 ACR/EULAR criteria.

### Ethical issues

This study was conducted in accordance with the Ethical Principles for Medical Research of the Declaration of Helsinki and was approved by the Clinical Research Ethics Committee of the Hospital Universitario 12 de Octubre (Reference numbers 18/009 and 18/182). Approval for the enrolment of the patients and sample shipment to the Hospital Universitario 12 de Octubre was obtained from the Ethics Committees of each center involved. An informed consent was also obtained from all patients enrolled in the study. Before the samples were sent, an anonymous code was assigned to each patient and serum sample at the hospital of origin to guarantee the anonymity. Anonymized clinical and analytical data from each center were integrated into a single database.

### Statistical methods

Clinical and demographic characteristics of patients were described by absolute frequency and percentage or median with the corresponding interquartile range (IQR).

Associations between qualitative variables were performed using Pearson’s X^2^ test or Fisher’s exact test, as appropriate. Wilcoxon-Mann-Whitney test (Mann-Whitney U-test) was used for comparisons of scaled and qualitative variables with two categories and Kruskal-Wallis’s test for comparisons of qualitative variables with more than two categories.

Odds ratio (OR) was used to measure the strength of association between the presence of a risk factor and a given outcome. The 95% confidence interval (95% CI) of OR was calculated by logistic regression or Miettinen-Nurminen method, as appropriate (36).

The box and whisker plots represent the values from the lower to upper quartile (from 25 to 75 percentile) in the central box. The median is represented as an inner line in the box. Probabilities <0.05 were considered significant.

Statistical analysis of data was performed using MedCalc Statistical Software version 19.5 (MedCalc Software, Ostend, Belgium).

## Results

### APS patients’ characteristics and prevalence of B2-CIC

A total of 303 patients classified as APS were included in the study from six European hospitals: Belgrade (N=119), Brescia (N=38), Florence (N=15), Madrid (N=29), Torino (N=66) and Warsaw (N=36). The median age of the cohort was 47 years (IQR: 38.0-57.0) and 220 patients (72.6%) were women (male:female ratio of 1:2.7) (**Supplementary Table S1**).

Clinical presentation. Thrombotic events alone were found in 196 patients (64.7%), 67 (22.1%) only had gestational morbidity and 40 (13.2%) had both thrombotic and obstetric manifestations. Of the total number of patients, 138 (45.5%) had SAD-APS (mainly associated with SLE, N=112, 37.0%) and 165 had primary APS (54.5%). In addition to SLE, other systemic autoimmune diseases present were RA (N=7), Sjögren’s syndrome (N=9) and systemic sclerosis (N=3). The main clinical characteristics of the cohort are shown in **Supplementary Table S2**.

Presence of aPL. Of the total patients, 146 (48.2%) were positive for IgG aB2GP1, 149 (49.2%) for IgM aB2GP1, 151 (49.8%) for IgG aCL and 132 (43.6%) for IgM aCL antibodies. LA was the most prevalent aPL, with 220 positive patients (72.6%) (**Table 1**). In relation to the presence of B2-CIC, 119 patients (39.3%) were positive for some B2-CIC; 35 (11.6%) were positive for B2G-CIC, 98 (32.3%) for B2M-CIC and 14 (4.6%) were positive for both (**Table 1**). The presence of B2-CIC was not detected in the serum sample in the remaining 184 patients (60.7%).

**Table 1:** Distribution of positive antiphospholipid antibodies and B2G/B2M-CIC in the 303 APS patients.

ANTIPHOSPHOLIPID ANTIBODIES	MULTICENTER COHORT OF APS PATIENTS (N=303)	
	Positive patients (N)	%
IgG aB2GP1	146	48.2
IgM aB2GP1	149	49.2
IgG aCL	151	49.8
IgM aCL	132	43.6
LA	220	72.6
B2G-CIC	35	11.6
B2M-CIC	98	32.3
B2-CIC	119	39.3
Triple aPL positivity	134	44.2
Quadruple aPL positivity	59	19.5

aB2GP1, anti-β2-glicoproteína-I antibodies; aCL, anti-cardiolipin antibodies; LA, lupus anticoagulant; B2G-CIC, immune-complexes formed by B2GP1 and IgG aB2GP1 antibodies; B2M-CIC, immune-complexes formed by B2GP1 and IgM aB2GP1 antibodies; B2-CIC, presence of B2G and/or B2M-CIC.

According to Guilford’s Rule of Thumb (37), correlations between the levels of aPL and B2-CIC showed a low-grade relationship in the case of B2G-CIC and IgG aCL (p=0.001, r=0.20) and IgG aB2GP1 antibodies (p<0.001, r=0.25). In patients with B2M-CIC, a moderate relationship with IgM aCL antibody levels (p<0.001, r=0.44) and a negligible relationship with IgM aB2GP1 antibodies (p=0.005, r=0.16) were observed.

Patients were differentiated according to their APS type. The following number of patients was positive for B2-CIC: 73/196 (37.2%) patients with isolated thrombotic APS, 35/67 (52.2%) with isolated gestational morbidity, 11/40 (27.5%) with mixed APS (both thrombotic and gestational manifestations), 84/236 (35.6%) of total thrombotic APS patients and 46/107 (43.0%) of total gestational APS patients (**Supplementary Table S3**).

### Clinical manifestations and laboratory parameters in patients positive for B2-CIC

Based on the presence or absence of B2-CIC (B2G and/or B2M-CIC), no differences were observed in distribution of sex, age, disease duration or proportion of primary APS between both groups. Regarding APS events, a higher percentage of women with isolated gestational morbidity positive for B2-CIC was observed compared to those who were negative (29.4% vs. 17.4%, p=0.014, OR: 1.98, 95% CI: 1.14-3.43) (**Table 2**).

**Table 2:** Clinical, demographic and laboratory parameters of the 303 APS patients grouped according to the positivity for B2-CIC.

CONDITION	B2-CIC positive N=119, 39.3%		B2-CIC negative N=184, 60.7%		p-value	OR/Hodges-Lehmann median difference (95% CI)
	N/median	%/IQR	N/median	%/IQR		
Age (years)	46.0	36.3-56.8	48.0	39.0-57.0	0.533	
Sex (women)	92	77.3	128	69.6	0.141	
Disease duration (years)	5.0	2.0-9.0	6.0	2.0-11.0	0.259	
Systemic lupus erythematosus	42	35.3	70	38.0	0.628	
Diabetes mellitus	7	9.1	15	13.0	0.458	
Arterial hypertension	23	19.3	39	21.2	0.694	
Dyslipidemia	24	20.2	34	18.5	0.716	
Active smoker	9	7.6	23	12.5	0.173	
Primary APS	64	53.8	101	54.9	0.850	
SAD-APS	55	46.2	83	45.1	0.850	
Catastrophic APS	1	0.8	7	3.8	0.154	
Isolated gestational morbidity	35	29.4	32	17.4	**0.014**	1.98 (1.14-3.43)
Isolated thrombotic APS	73	61.3	123	66.8	0.329	
Inf. extr. deep vein thrombosis	35	29.4	58	31.5	0.521	
Superficial thrombophlebitis	19	16.0	23	12.5	0.394	
Sup. extr. arterial thrombosis	1	0.8	8	4.3	0.083	
Acute myocardial infarction	6	5.0	8	4.3	0.779	
Stroke	22	18.5	42	22.8	0.366	
Pulmonary embolism	8	6.7	21	11.4	0.176	
Chorea	3	2.5	3	1.6	0.683	
Epilepsy	4	3.4	14	7.6	0.143	
Migraine	16	13.4	28	15.2	0.670	
Transient global amnesia	4	3.4	2	1.1	0.216	
Cephalea	3	2.5	6	3.3	1.000	
Unstable angina	1	0.8	6	3.3	0.252	
Chronic cardiomyopathy	0	0.0	2	1.1	0.521	
Vegetations	9	7.6	20	10.9	0.340	
Pseudoinfective endocarditis	4	3.4	8	4.3	0.770	
Valve thickening and dysfunction	5	4.2	1	0.5	**0.036**	8.03 (1.22-52.45)
Primary pulmonary hypertension	1	0.8	0	0.0	0.393	
Secondary pulmonary hypertension	3	2.5	1	0.5	0.304	
Major pulmonary arterial thrombosis	2	1.7	2	1.1	0.647	
Pulmonary microthrombosis	5	4.2	16	8.7	0.133	
Glomerular capillary thrombosis	0	0.0	1	0.5	1.000	
Renal artery trunk lesions	0	0.0	2	1.1	0.521	
Renal vein thrombosis	1	0.8	0	0.0	0.393	
Livedo reticularis	26	21.8	28	15.2	0.141	
Skin ulcerations	7	5.9	11	6.0	0.973	
Pseudovasculitic lesions	12	10.1	23	12.5	0.521	
Superficial cutaneous necrosis	3	2.5	5	2.7	1.000	
Amaurosis fugax	0	0.0	2	1.1	0.521	
Retinal artery thrombosis	3	2.5	1	0.5	0.304	
Optic neuropathy	1	0.8	6	3.3	0.251	
Ophthalmic sicca	12	10.1	18	9.8	0.931	
Thrombocytopenia	32	26.9	35	19.0	0.107	
Autoimmune haemolytic anemia	14	11.8	14	7.6	0.223	
Microangiopathic haemolytic anemia	4	3.4	2	1.1	0.216	
Leucopenia	18	15.1	30	16.3	0.784	
Disseminated intravascular coagulation	2	1.7	4	2.2	1.000	
C3 levels (mg/dL)	113.0	92.5-138.0	137.0	109.0-167.0	**<0.001**	23.0 (13.0-33.0)
C4 levels (mg/dL)	20.6	14.4-28.1	27.8	18.3-37.7	**<0.001**	6.39 (3.10-9.60)
Hypocomplementemia	32	26.9	36	19.6	0.167	
Platelets (x10^3^/μL)	216.0	158.8-260.0	234.5	198.0-277.0	**0.022**	23.0 (4.0-44.0)
IgG aCL positive	68	57.1	83	45.4	**0.038**	1.64 (1.03-2.61)
IgM aCL positive	62	52.1	70	38.0	**0.015**	1.78 (1.12-2.86)
IgG B2GP1 positive	62	52.1	84	45.7	0.273	
IgM B2GP1 positive	62	52.1	87	47.3	0.413	
LA positive	87	73.1	133	72.3	0.875	
Triple aPL positivity	59	49.6	75	40.8	0.132	
B2G-CIC positive	35	29.4	Na	Na	Na	
B2M-CIC positive	98	82.4	Na	Na	Na	
Antiplatelet agents	77	64.7	129	70.1	0.299	
Anticoagulants	108	90.8	161	87.5	0.350	
Treated	112	94.1	170	92.4	0.519	

Significant p-values <0.05 are represented in bold.

No differences were observed in the incidence of clinical manifestations associated with APS from different medical specialties, except for a higher incidence of heart valve thickening and dysfunction in B2-CIC-positive patients compared to those who were negative (4.2% vs. 0.5%, p=0.036, OR: 8.03, 95% CI: 1.22-52.45) (**Table 2**).

**Figure 1:**
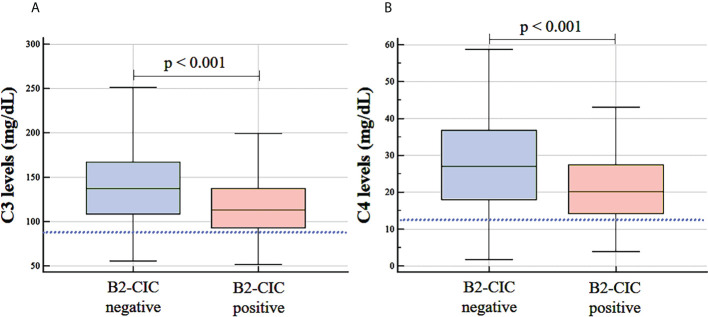
Levels of complement factors C3 **(A)** and C4 **(B)** in B2-CIC-positive and negative patients (N=303). The lower limit of the normal range for C3 (88 mg/dl) and C4 levels (12 mg/dl) is represented by dotted lines.

In terms of laboratory parameters, although mean C3 and C4 values were within the normal range in both groups, the levels were significantly lower in B2-CIC-positive patients compared to negative patients: C3 levels (median: 113.0 mg/dL, IQR: 92.5-138.0 vs. median: 137.0 mg/dL, IQR: 109.0-167.0, p<0.001, Hodges-Lehmann median difference: 23.0, 95% CI: 13.0-33.0) and C4 levels (median: 20.6 mg/dL, IQR: 14.4-28.1 vs. median: 27.8 mg/dL, IQR: 18.3-37.7, p<0.001, Hodges-Lehmann median difference: 6.39, 95% CI: 3.10-9.60) (**Figure 1**; **Table 2**). Levels of complement activation products C3a and C5a were measured in a sample of 51 patients. B2-CIC-positive patients had higher C3a levels compared to the negative ones (mean: 1.98 ng/mL, IQR: 0.87-5.20 vs. mean: 0.82 ng/mL, IQR: 0.30-4.47), but these differences were very close to reaching significance (p=0.059). No differences were observed in the mean levels of C5a between B2-CIC-positive and negative patients (1.73 ng/mL vs. 1.87 ng/mL, respectively, p=0.823).

Additionally, a significant decrease in platelet count was observed in patients positive for B2-CIC compared to the negative ones (median: 216.0 x10^3^/μL, IQR: 158.8-260.0 vs. median: 234.5 x10^3^/μL, IQR: 198.0-277.0, p=0.022, median Hodges-Lehmann difference: 23.0, 95% CI: 4.0-44.0). However, this was not associated with a higher incidence of thrombocytopenia in the total cohort (**Table 2**).

Furthermore, a higher prevalence of IgG aCL antibodies was observed in B2-CIC-positive patients than in those negative (57.1% vs. 45.4%, p=0.038, OR: 1.64, 95% CI: 1.03-2.61), in addition to a higher prevalence of IgM aCL antibodies (52.1% vs. 38.0%, p=0.015, OR: 1.78, 95% CI: 1.12-2.86) (**Table 2**).

### Evaluation of antiphospholipid immune-complexes by classical methodologies

To demonstrate that immune-complexes precipitated by PEG-6000 were formed by B2GP1, a capture ELISA of the precipitates was performed. Six patients with thrombotic APS and positive for B2G-CIC were tested, as well as 2 blood donors as controls. Polyethylene glycol precipitates from all six patients contained B2GP1 (mean: 2.357 OD vs. 0.096 OD in controls). In addition, the presence of IgG and the incorporation of complement factor C1q into B2-CIC was confirmed in all six precipitates but not in controls (mean: 1.113 OD vs. 0.178 OD in controls) (**Supplementary Figure S1** **)**.

### Immune-complexes stability

The same six APS patients positive for B2-CIC and the two controls evaluated by PEG-6000 were also studied to determine whether new B2-CIC could be formed during the time of the assay. We observed that purified IgG from B2-CIC-positive patients was unable to bind to the purified human B2GP1 (>99% in a closed conformation in plasma), with OD values similar to those of the negative control (mean: 0.020 OD and 0.026 OD, respectively). However, the anti-human B2GP1 monoclonal antibody used as a positive control was able to detect the presence of the protein in the assay (mean: 2.726 OD).

The ability of purified IgG from the six B2G-CIC-positive patients to recognize the open conformation of B2GP1 was tested using a commercial assay designed to determine aB2GP1 antibodies. Mean levels of IgG aB2GP1 antibodies of patients were 67 U/ml. The lowest value was 23 U/ml, which was above the cut-off point considered by the manufacturer (10 U/ml). These results suggest that patient sera are able to recognize the open form of B2GP1 but not the closed conformation in plasma.

### Characteristics of patients with thrombotic APS and B2-CIC

Considering the 236 patients who had suffered thrombotic events (total thrombotic APS, without excluding patients who additionally had some obstetric symptoms), no significant differences were observed in sex, age, disease duration, presence of cardiovascular risk factors, proportion of primary APS or incidence of most APS-related clinical manifestations between positive and negative patients for B2-CIC, except for a higher incidence of heart valve thickening and dysfunction in B2-CIC-positive patients compared to the negative patients (6.0% vs. 0.7%, p=0.015, OR: 9.60, 95% CI: 1.44-62.71) (**Supplementary Table S4**).

**Figure 2:**
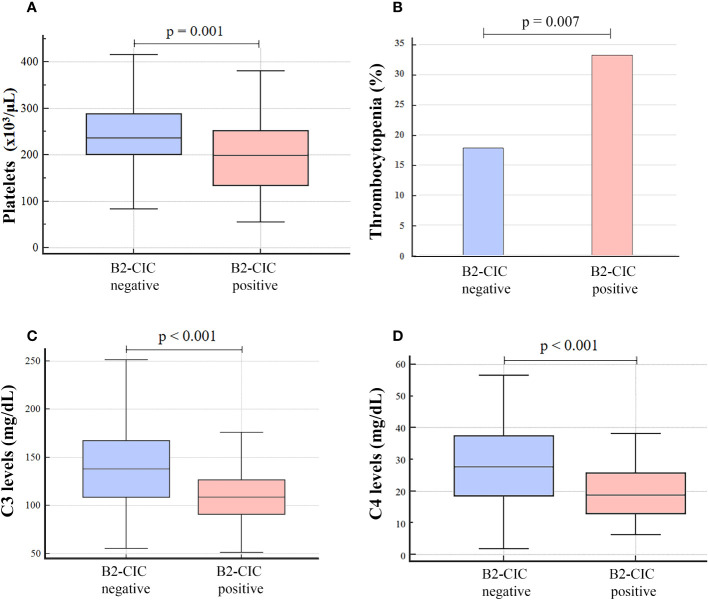
Platelet count **(A)**, incidence of thrombocytopenia **(B)** and levels of C3 **(C)** and C4 **(D)** in patients with total thrombotic APS, positive and negative for B2-CIC (N=236).

A significant decrease in platelet count was observed in B2-CIC-positive patients in comparison with those who were negative (median: 199.5 x10^3^/μL, IQR: 135.0-252.0 vs. median: 235.5 x10^3^/μL, IQR: 200.0-288.0, p=0.001, median Hodges-Lehmann difference: 42.0, 95% CI: 16.0-68.0) in addition to a higher incidence of thrombocytopenia (33.3% vs. 17.8%, p=0.007, OR: 2.32, 95% CI: 1.25-4.28) (**Figures 2A, B**; **Supplementary Table S4**). Besides, a significant reduction in the complement factors levels was observed in patients positive for B2-CIC compared to the negative ones: C3 levels (median: 109.0 mg/dL, IQR: 91.0-129.0 vs. median: 138.5 mg/dL, IQR: 109.0-167.0, p<0.001, Hodges-Lehmann median difference: 29.0, 95% CI: 18.0-41.0) and C4 levels (median: 18.9 mg/dL, IQR: 13.0-26.8 vs. median: 27.7 mg/dL, IQR: 18.6-37.4, p<0.001, Hodges-Lehmann median difference: 7.90, 95% CI: 4.20-11.88) (**Figures 2C, D**; **Supplementary Table S4**).

A higher prevalence of IgG aCL antibodies was observed in B2-CIC-positive patients compared to the B2-CIC negative patients (64.3% vs. 46.1%, p=0.005, OR: 2.18, 95% CI: 1.26-3.79) in addition to a higher incidence of triple aPL positivity (57.1% vs. 42.1%, p=0.027, OR: 1.83, 95% CI: 1.07-3.14) (**Supplementary Table S4**).

### Characteristics of patients with isolated thrombotic APS and B2-CIC

When the 196 patients with isolated thrombotic APS were considered, we observed the same pattern as in the previous group. No differences were observed in age, sex, disease duration, proportion of primary APS or presence of most clinical manifestations associated with APS between B2-CIC-positive and negative patients. However, a higher incidence of heart valve thickening was observed in patients positive for B2-CIC compared to those who were negative (5.5% vs. 0.8%, p=0.047, OR: 7.07, 95% CI: 1.03-47.97**)** (**Table 3**).

**Table 3:** Clinical, demographic and laboratory parameters of patients with isolated thrombotic APS, positive and negative for B2-CIC (N=196).

ISOLATED THROMBOTIC APS (N=196)						
CONDITION	B2-CIC positive N=73, 37.2%		B2-CIC negative N=123, 62.8%		p-value	OR/Hodges-Lehmann Median Difference (95% CI)
	N/median	%/IQR	N/median	%/IQR		
Age (years)	54.0	41.0-61.3	50.0	40.3-58.0	0.205	
Sex (women)	46	63.0	67	54.5	0.243	
Disease duration (years)	5.0	3.0-10.0	5.0	2.0-10.0	0.393	
Systemic lupus erythematosus	30	41.1	51	41.5	0.960	
Primary APS	36	49.3	63	51.2	0.797	
SAD-APS	37	50.7	60	48.8	0.797	
Catastrophic APS	0	0.0	4	3.3	0.299	
Arterial thrombosis	32	43.8	63	51.2	0.319	
Venous thrombosis	43	58.9	65	52.8	0.170	
Inf. extr. deep vein thrombosis	31	42.5	50	40.7	0.455	
Superficial thrombophlebitis	15	20.5	18	14.6	0.286	
Sup. extr. arterial thrombosis	1	1.4	6	4.9	0.239	
Acute myocardial infarction	4	5.5	5	4.1	0.729	
Stroke	20	27.4	36	29.3	0.780	
Pulmonary embolism	7	9.6	13	10.6	0.827	
Valve thickening and dysfunction	4	5.5	1	0.8	**0.047**	7.07 (1.03-47.97)
Thrombocytopenia	22	30.1	20	16.3	**0.022**	2.22 (1.11-4.44)
Hypocomplementemia	24	32.9	28	22.8	0.141	
C3 levels (mg/dL)	109.0	90.0-130.0	135.5	108.5-161.5	**<0.001**	27.0 (15.0-39.0)
C4 levels (mg/dL)	18.8	13.0-25.8	26.2	17.2-35.5	**0.001**	7.10 (3.30-11.10)
Platelets (x10^3^/μL)	198.0	130.0-253.3	244.0	203.3-292.3	**0.001**	46.0 (21.0-76.0)
IgG aCL positive	47	64.4	57	46.3	**0.011**	2.18 (1.19-3.97)
IgM aCL positive	35	47.9	45	36.6	0.100	
IgG B2GP1 positive	43	58.9	56	45.5	0.071	
IgM B2GP1 positive	36	49.3	53	43.1	0.399	
LA positive	57	78.1	88	71.5	0.314	
B2G-CIC positive	26	35.6	Na	Na	Na	
B2M-CIC positive	58	79.5	Na	Na	Na	
Triple aPL positivity	43	58.9	49	39.8	**0.010**	2.17 (1.20-3.90)
Antiplatelet agents	35	47.9	78	63.4	**0.028**	0.51 (0.28-0.93)
Anticoagulants	64	87.7	106	86.2	0.817	
Treated	68	93.2	111	90.2	0.477	

aB2GP1, anti-β2-glicoproteína-I antibodies; aCL, anti-cardiolipin antibodies; LA, lupus anticoagulant; B2G-CIC, immune-complexes formed by B2GP1 and IgG aB2GP1 antibodies; B2M-CIC, immune-complexes formed by B2GP1 and IgM aB2GP1 antibodies. Significant p-values <0.05 are represented in bold.

**Figure 3:**
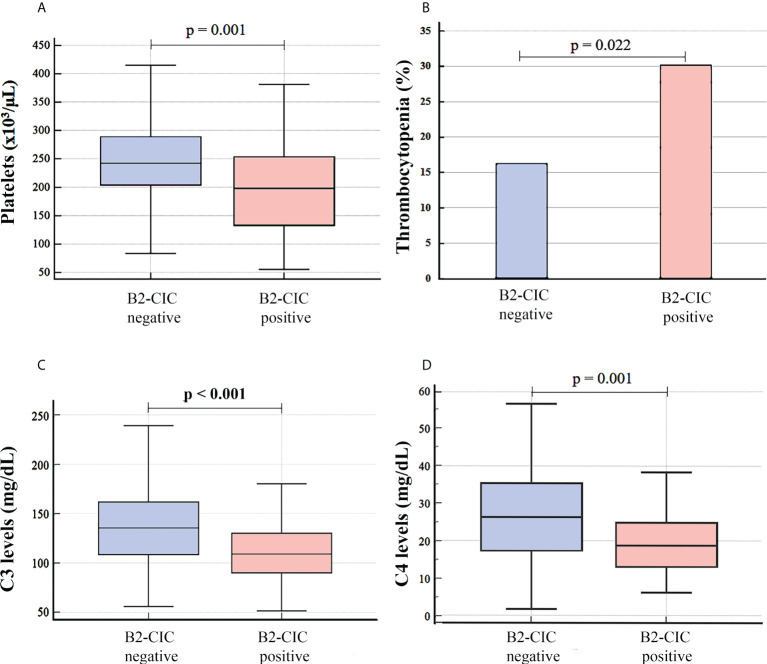
Platelet count **(A)**, incidence of thrombocytopenia **(B)** and levels of complement factor C3 **(C)** and C4 **(D)** in patients with isolated thrombotic APS, differentiating between B2-CIC-positive and negative patients (N=196).

A decrease in the platelet count was observed in patients with B2-CIC in relation to patients without these (median: 198.0 x10^3^/μL, IQR: 130.0-253.3 vs. median: 244.0 x10^3^/μL, IQR: 203.3-292.3, p=0.001, Hodges-Lehmann median difference: 46.0, 95% CI: 21.0-76.0) and this was associated with a higher incidence of thrombocytopenia (30.1% vs. 16.3%, p=0.022, OR: 2.22, 95% CI: 1.11-4.44) (**Figures 3A, B**; **Table 3**).

Lower levels of complement factors were also observed in patients positive for B2-CIC versus those who were negative: C3 levels (median: 109.0 mg/dL, IQR: 90.0-130.0 vs. median: 135.5 mg/dL, IQR: 108.5-161.5, p<0.001, Hodges-Lehmann median difference: 27.0, 95% CI: 15.0-39.0) and C4 levels (median: 18.8 mg/dL, IQR: 13.0-25.8 vs. median: 26.2 mg/dL, IQR: 17.2-35.5, p=0.001, Hodges-Lehmann median difference: 7.10, 95% CI: 3.30-11.10) (**Figures 3C, D**; **Table 3**).

A higher prevalence of IgG aCL antibodies was observed in B2-CIC-positive patients compared to those who were negative (64.4% vs. 46.3%, p=0.011, OR: 2.18, 95% CI: 1.19-3.97), as well as a higher prevalence of triple aPL positivity (58.9% vs. 39.8%, p=0.010, OR: 2.17, 95% CI: 1.20-3.90**)** (**Table 3**).

In addition, a lower proportion of B2-CIC-positive patients with isolated thrombotic APS were treated with antiplatelet therapy compared to the negative ones (47.9% vs. 63.4%, p=0.028, OR: 0.51, 95% CI: 0.28-0.93), although no differences in terms of anticoagulant therapy or proportion of treated patients were observed (**Table 3**).

### Characteristics of patients with gestational morbidity and B2-CIC

Taking into account the 107 patients with gestational morbidity (without excluding those who had suffered an additional thrombotic event), no significant differences were observed in the main clinical characteristics or presence of manifestations associated with APS between B2-CIC positive and negative patients. A shorter disease duration (years since the diagnosis) was observed in the case of B2-CIC-positive patients compared to the negative ones (median: 3.0 years, IQR: 1.6-7.3 vs. median: 8.0 years, IQR: 4.0-14.0, p=0.002, Hodges-Lehmann median difference: 3.0, 95% CI: 1.0-6.0).

In the 40 patients with mixed APS (both thrombotic events and gestational morbidity), no differences were observed between B2-CIC-positive and negative patients for the main clinical characteristics and laboratory parameters evaluated. As occurred previously, only a shorter disease duration was observed in B2-CIC-positive patients compared to the negative ones (median: 4.0 years, IQR: 2.0-6.8 vs. median: 8.5 years, IQR: 4.0-17.6, p=0.021, Hodges-Lehmann median difference: 4.5, 95% CI: 1.0-11.9).

### Characteristics of patients with isolated gestational morbidity and B2-CIC

In the 67 patients with isolated gestational APS, no significant differences were observed in age, presence of clinical manifestations related to APS, disease duration, laboratory parameters or treatment received between the B2-CIC-positive and negative patients.

### Differences between isolated thrombotic and gestational APS patients

To determine whether the previous differences were observed due to the syndrome’s clinical presentation rather than the presence of B2-CIC, we compared the previously identified significant variables (p<0.050) between the three main groups of patients according to the clinical events they suffered (independently of B2-CIC positivity): isolated thrombotic APS, isolated gestational morbidity and mixed APS.

Patients with isolated gestational morbidity were younger (median: 39.0 years, IQR: 32.0-44.0) than patients with mixed APS (median: 49.0 years, IQR: 37.5-56.5, p=0.003) and isolated thrombotic APS (median: 51.5 years, 41.0-59.0, p<0.001) (**Supplementary Table S5**). In addition, a higher proportion of patients with isolated gestational morbidity were treated with antiplatelet agents (91.0%) compared to patients with mixed APS (80.0%, p=0.033) and isolated thrombotic APS (57.7%, p<0.001). This difference was also observed between patients with mixed APS and isolated thrombotic APS (p=0.015) (**Supplementary Table S5**).

Higher B2M-CIC levels were observed in patients with isolated gestational morbidity (median: 19.0 U/mL, IQR: 8.2-30.8) compared to patients with isolated thrombotic APS (median: 11.0 U/mL, IQR: 5.8-22.1, p=0.019), but not when compared with mixed APS (median: 10.1 U/mL, IQR: 5.9-24.0, p=0.089). No differences were observed between patients with isolated thrombotic and mixed APS (p=0.990). The difference observed in B2M-CIC levels resulted in a higher prevalence of B2M-CIC-positive patients with isolated gestational morbidity (44.8%) in comparison with patients with isolated thrombosis (29.6%, p=0.023) and mixed APS (25.0%, p=0.042) (**Supplementary Table S5**).

Lastly, higher B2G-CIC levels were observed in patients with isolated thrombotic APS (median: 5.6 U/mL, IQR: 3.1-10.5) compared to patients with isolated gestational morbidity (median: 3.8 U/mL, IQR: 2.0-8.2, p=0.013) and mixed APS (median: 4.2 U/mL, IQR: 2.2-6.0, p=0.015). No difference was observed between patients with mixed APS and isolated gestational morbidity (p=0.750). However, the difference found in the levels did not imply a significant difference in the prevalence of B2G-CIC-positive patients (**Supplementary Table S5**).

**Figure 4:**
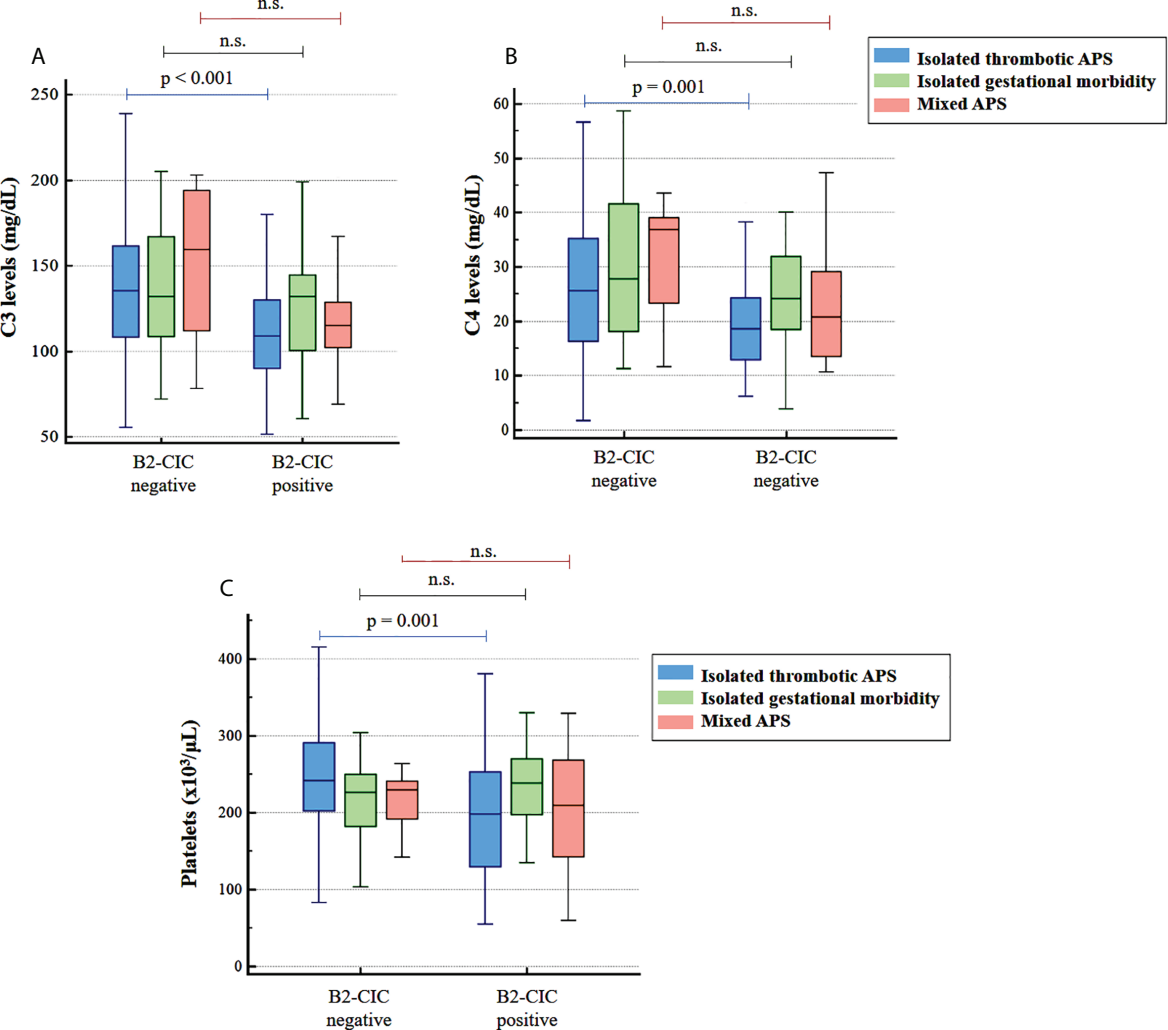
Levels of C3 **(A)**, C4 **(B)** and platelets **(C)** in patients with isolated thrombotic APS (blue), isolated gestational morbidity (green) and mixed APS (red) according to the positivity for B2-CIC. ns, not significant.

Statistically significant differences were observed in the lower levels of C3, C4 and platelets in B2-CIC-positive patients with isolated thrombotic APS compared to the negative patients as well as in the higher prevalence of thrombocytopenia only when patients were differentiated according to the positivity for B2-CIC (**Figure 4**; **Table 3**). These differences were not observed in patients with isolated gestational morbidity or mixed APS (**Figure 4**).

Finally, patients with B2-CIC and isolated thrombotic APS were compared to B2-CIC-positive patients with isolated gestational morbidity. Patients with thrombotic APS were older than gestational patients (median: 54.0 years, IQR: 41.0-61.3 vs. median: 38.0 years, IQR: 33.3-41.8, p<0.001, Hodges-Lehmann median difference: 15.0, 95% CI: 9.0-19.0) and had a higher prevalence of triple aPL positivity (58.9% vs. 31.4%, p=0.009, OR: 3.13, 95% CI: 1.33-7.34) (**Table 4**). Regarding complement factors, patients with thrombotic APS had lower C3 levels compared to patients with gestational morbidity (median: 109.0 mg/dL, IQR: 90.0-130.0 vs. median: 132.0 mg/dL, IQR: 100.5-144.8, p=0.028, Hodges-Lehmann median difference: 19.0, 95% CI: 2.0-34.0), in addition to lower C4 levels (median: 18.8 mg/dL, IQR: 13.0-25.8 vs. median: 24.2 mg/dL, IQR: 18.5-31.9, p=0.016, Hodges-Lehmann median difference: 5.70, 95% CI: 1.20-10.30) (**Table 4**). Additionally, a decrease in the platelet count was observed in patients with thrombosis compared to those with obstetric manifestations (median 198.0 x10^3^/μL, IQR: 130.0-253.3 vs. median: 239.0 x10^3^/μL, IQR: 197.5-270.5, p=0.025, Hodges-Lehmann median difference: 36.0, 95% CI: 5.0-67.0), resulting in a higher incidence of thrombocytopenia (30.1% vs. 11.4%, p=0.034, OR: 3.34, 95% CI: 1.05-10.61) (**Table 4**).

**Table 4:** Comparison of clinical and laboratory characteristics previously identified with a p-value <0.050 between B2-CIC-positive patients with isolated thrombotic APS and B2-CIC-positive patients with isolated gestational morbidity.

CONDITION	ISOLATED THROMBOTIC APS B2-CIC + (N=73, 37.2%)	ISOLATED GESTATIONAL APS B2-CIC + (N=35, 52.2%)	p-value	OR/Hodges-Lehmann Median Difference (95% CI)
	N (%)/median (IQR)	N (%)/median (IQR)		
Age (years)	54.0 (41.0-61.3)	38.0 (33.3-41.8)	**<0.001**	15.0 (9.0-19.0)
Platelets (x10^3^/μL)	198.0 (130.0-253.3)	239.0 (197.5-270.5)	**0.025**	36.0 (5.0-67.0)
Thrombocytopenia	22 (30.1)	4 (11.4)	**0.034**	3.34 (1.05-10.61)
C3 levels (mg/dL)	109.0 (90.0-130.0)	132.0 (100.5-144.8)	**0.028**	19.0 (2.0-34.0)
C4 levels (mg/dL)	18.8 (13.0-25.8)	24.2 (18.5-31.9)	**0.016**	5.70 (1.20-10.30)
Valve thickening and dysfunction	4 (5.5)	0 (0.0)	0.160	
Triple aPL positivity	43 (58.9)	11 (31.4)	**0.009**	3.13 (1.33-7.34)
Disease duration (years)	5.0 (3.0-10.0)	3.0 (1.5-9.0)	0.079	

Significant p-values <0.05 are represented in bold.

The same statistically significant differences were obtained when B2-CIC-positive patients with total thrombotic APS were compared with B2-CIC-positive patients with isolated gestational morbidity (data not shown).

## Discussion

The presence of B2G and B2M-CIC in a cohort of APS patients having different geographical origins has been confirmed in this multicenter study. B2-CIC presence in patients with thrombotic antecedents was significantly associated with a decrease of the platelet count and complement factors C3 and C4 levels as well as with a higher incidence of thrombocytopenia, thus confirming what has been observed in previous single-center studies (26–28).

In APS patients, the development of thrombotic events is not always triggered by the presence of aPL. Therefore, additional biomarkers are needed to support the disease assessment. In regards to this, the presence of B2A-CIC has been described in patients with thrombotic manifestations of APS (24, 25). A prevalence of 39.3% for B2-CIC formed by aB2GP1 antibodies of IgG or IgM isotypes was observed in this multicenter study performed on a cohort of 303 patients with a diagnosis of APS. Patients with B2-CIC and thrombotic APS had a higher incidence of thrombocytopenia, heart valve thickening, and triple aPL positivity compared to the B2-CIC negative patients. Although the mean levels of C3 and C4 in patients with B2-CIC were within the normal ranges, both were significantly lower than in patients without B2-CIC. These differences were not observed in patients with mixed APS or isolated gestational morbidity.

In order to confirm whether the differences found between patients with and without B2-CIC were observed due to the type of APS presented rather than to the presence of B2-CIC, a comparison of the patients was conducted only in regards to the clinical events they suffered from. Patients with isolated gestational morbidity were younger than patients with mixed and isolated thrombotic APS, coinciding with an obvious younger age for women of fertile age. In addition, they were more frequently treated with antiplatelet therapy compared to the two other groups. This is related to the standard therapy received for treating each type of event, since low-dose aspirin is recommended for non-pregnant women with a history of obstetric APS and also during pregnancy together with heparin, while the baseline treatment for venous and arterial thrombosis is mainly by anticoagulation (VKA) (38). Furthermore, higher levels of B2M-CIC were observed in patients with isolated gestational morbidity compared to patients with isolated thrombotic APS, but not with mixed APS (probably due to the low number of patients in this group). This difference in the levels resulted in a higher prevalence of B2M-CIC-positive patients with isolated gestational morbidity in relation to patients with thrombosis.

It stands out that there was a significantly higher proportion of B2M-CIC-positive patients among those with obstetric APS without a history of thrombosis (isolated gestational APS). This finding is in agreement with the known fact that the IgM isotype is important in obstetric APS: isolated IgM aPL are frequent in obstetric APS and rare in thrombotic APS (39). This reinforces the idea that obstetric and thrombotic APS most likely have a different pathogenesis despite sharing the same antigenic specificity of the antibodies (40).

Under physiological conditions, B2GP1 is only present in the decidual endothelium and placental tissues but not in the endothelium of the rest of the body, where is only detected after an inflammatory stimulus (41, 42). The placental damage found during pregnancy could be explained because the large amount of B2GP1 would be recognized by aPL in both free form and bound to B2GP1, forming B2-CIC. Since IgM is the most efficient isotype activating complement (43), the pathogenic role of B2M-CIC could be mediated by the complement. The role of complement in obstetric APS is well known as the deposition of complement factors has been described in both the placentas of gestational-APS patients and in animal models of this illness. Furthermore, the infusion of complement-blocking molecules has been shown to protect against fetal loss in these pregnant mice APS models (44–46).

On the contrary, although patients with isolated thrombotic APS had higher levels of B2G-CIC compared to patients with isolated gestational morbidity and mixed APS, there were no differences in the prevalence of B2G-CIC-positive patients among the three groups.

Our results show that the type of APS event was not associated to the decrease of platelet and complement factor levels between patients positive and negative for B2-CIC. The comparison of B2-CIC-positive patients with isolated thrombotic APS (as well as patients with total thrombotic APS) and B2-CIC-positive patients with isolated gestational morbidity confirmed that patients with thrombotic manifestations and positive for B2-CIC had lower levels of C3, C4 and platelets as well as an increased incidence of thrombocytopenia and triple positivity compared to patients with obstetric APS. The higher C3a levels in patients with B2-CIC, although not significant, could suggest a tendency towards an increased complement activation in these patients. The nearly significant and non-significant association of C3a and C5a levels with the presence of B2-CIC, respectively, may be due to the short half-life of these complement activation products [a half-life of 30 minutes for C3a and 5 minutes for C5a has been described (47–49)]. Although patients with isolated gestational morbidity had a higher prevalence of B2-CIC, this did not result in an alteration of complement factors or platelets. However, patients with thrombotic APS had a higher complement and platelet consumption, suggesting a higher degree of platelet activation leading to an increase of thrombocytopenia in B2-CIC-positive patients. In the case of complement factors, the consumption was moderate and although there was a decrease in the total levels of C3 and C4, an increased percentage of patients with hypocomplementemia was not found.

A clear correlation was not observed in the evaluation of circulating levels of aPL and B2-CIC. The aPL evaluation assays can only detect the free forms of antibodies but not aPL bounded to their antigen. In this way, B2-CIC-positive patients may have low levels of circulating aPL or even negative results because the antibodies integrated in B2-CIC could not be detected, so the observed aPL levels may be lower than those prior to the B2-CIC formation (30, 50). Only low-affinity antibodies would remain not attached to their antigen and thus could be detected by conventional assays. This could be a paradoxical situation in which the presence of high-affinity antibodies could predominate in patients in whom the detected aPL titers by conventional assays are below the cutoff. Future studies evaluating this possibility would be of interest.

We have verified that purified immunoglobulins from B2G-CIC-positive patients are not able to recognize the purified B2GP1 from human serum (>99% in a circular conformation under physiological conditions). However, these purified immunoglobulins can bind to B2GP1 when it is in an open form (easily detected by commercial assays for the determination of IgG aB2GP1 antibodies), confirming our suspicions that B2-CIC can only be formed after B2GP1 changes its conformation to the open form, a situation that only occurs after its activation. These results are in agreement with Meroni’s two-hit hypothesis: the presence of aPL is not sufficient to develop thrombotic events because B2GP1 circulates in a closed conformation, so a second hit that activates the endothelium is needed (14). B2GP1 binds to activated endothelial cells, opens its conformation and exposes hidden epitopes enabling the binding of aPL. The B2GP1-aPL binding is stable and can only dissociate under conditions involving extreme pH shocks or after numerous cycles of freezing and thawing (25). The presence of B2-CIC could indicate that, at some moment, a second hit could have triggered the conformational change of B2GP1 to the open form, allowing the antigen-antibody binding. In this sense, the presence of B2-CIC maybe could be a surrogate marker for the action of a second hit, triggering the protein opening and allowing aPL binding. This hypothesis has to be confirmed by future studies.

The presence of B2-CIC in APS was described in 1995 in a patient with catastrophic APS by heparin affinity chromatography (51). The use of this challenging technology was hampered in the clinical practice and the presence of B2-CIC has been poorly studied up to date. In this study, we have used an easily reproducible methodology to detect the presence of B2G and B2M-CIC that could be implemented in a simple manner in diagnostic laboratories. Using this method, our group have recently described the presence of B2A-CIC (24, 25), B2G-CIC and B2M-CIC (28) in APS patients. They were associated with an increased risk of thrombosis or presence of clinical manifestations related to APS such as thrombocytopenia and livedo reticularis (26–28).

It is well known that the APS clinical spectrum goes beyond the classification criteria and there are other clinical events strongly associated with APS such as thrombocytopenia (11, 52). In this multicenter cohort, thrombocytopenia is the most frequently observed non-criteria clinical manifestation, with a prevalence of 22% in the total cohort and 33.3% in B2-CIC-positive patients with thrombotic APS. It is similar to the estimated prevalence in other studies (20-53%) (7). In the study of 1000 APS patients performed by the Euro-Phospholipid Project Group, thrombocytopenia was the most frequent non-criteria clinical manifestation observed (29.6%). If clinical classification criteria are also considered, thrombocytopenia would be the second most frequent manifestation found after deep vein thrombosis (53). After a 10-year follow-up of these APS patients, the most common manifestations observed (including both criteria and non-criteria) were thrombocytopenia, livedo reticularis, stroke and gestational morbidity (12). Some authors have proposed that patients with thrombocytopenia and positive aPL represent a subgroup of patients with a pro-thrombotic state that precedes the onset of APS (52, 54, 55). Following the 14th International Congress on Antiphospholipid Antibodies, the task force recommended the inclusion of manifestations such as thrombocytopenia in the revision of classification criteria (56). The role of aPL in the pathogenesis of thrombocytopenia is not clearly understood. It has been postulated that these autoantibodies bind to activated platelets through the interaction of B2GP1 to glycoprotein Ibα (GPIbα) and ApoER2 receptors (57, 58). After the binding of aB2GP1/B2GP1 immune-complexes to these receptors, platelet activation *via* p38MAPK pathway was observed (59, 60). This platelet activation led to a subsequent endothelial cell activation and fibrin generation (61, 62). This mechanism could explain the thrombocytopenia found in patients with B2-CIC. Using murine models of APS, thrombocytopenia is developed as one of the most relevant features of the disease when mice are immunized with purified B2GP1 protein (63, 64).

In APS, the antigen (B2GP1) and the aB2GP1 antibody that recognizes it coexist in blood, but the occurrence of thrombosis is not always induced. Therefore, there must be more than one mechanism involved in the pathogenesis of APS. The presence of circulating B2-CIC could be a new biomarker related to the presence of the protein B2GP1 circulating in an open or “hook” conformation that could be recognized by aPL present in blood due to the exposure of epitopes previously hidden in the closed conformation that were not accessible by aPL (65, 66). The presence of open forms of B2GP1 would imply that aPL could be interacting with the soluble protein but also with this form of B2GP1 associated to membrane receptors such as GPIbα, ApoER2, annexin A2 or toll-like receptors (previously described as B2GP1-associated molecules) (15). The stimulation of these receptors by aPL initiates the activation of endothelial cells and could activate the complement pathway causing inflammatory lesions (66).

The complement system plays an important role in the clearance of pathogens and apoptotic cells and it is an important component of the immune response (67). We have observed lower levels of C3 and C4 in B2-CIC-positive patients with thrombotic APS, suggesting a higher degree of complement consumption. Moreover, it has been confirmed that the detected immune-complexes are formed by B2GP1 and IgG aPL, and they can incorporate complement factor C1q. IgG and IgM antibodies present in B2-CIC could lead to an increased activation of the classical pathway of complement (68, 69) as well as the lectin pathway (70). Clinical and experimental data accumulated in recent years support the role of the complement system as a key factor in the pathogenesis of APS (44, 71). A reduction in C3 and C4 levels (in addition to an increase of their activation products C3a and C4a) has been described in patients with vascular APS (72, 73), suggesting that the decrease in the complement levels is due to their consumption and activation of complement pathway (74). However, no association with serological parameters has been found. Moreover, some studies have described deposits of Ig, C1q and C3 in the heart valves of patients with aPL-associated valvulopathy (75), in renal biopsies of patients with APS nephropathy (76) and in a male patient who underwent bypass surgery after which he developed arterial thrombosis (77). Therefore, in addition to serological consumption, these findings suggest that complement activation also takes place at the tissue level. The critical role of the complement in mediating the damaging effects of aPL has also been studied using animal models of APS. Blockade of its activation by neutralizing antibodies prevented the clots formation and endothelial cell activation and aPL failed to exert their pro-coagulant effect in C3 and C5 deficient mice (78, 79).

Patients with catastrophic APS or multiple arterial thrombosis refractory to standard therapy have benefited from the use of medications such as eculizumab (80–82). However, chronic administration of this treatment to prevent thrombotic events would have a high cost, so it would be restricted to situations in which the development of coagulation is more probable. In this respect, the evaluation of B2-CIC could be a biomarker to assess the disease activity in APS patients, indicating those with a higher complement consumption who could benefit from the administration of this therapy. Further studies are needed to evaluate this possible hypothesis and to confirm the association with complement consumption in patients with thrombotic events.

A predominant presence of B2M-CIC (32.3%) has been observed as regards the prevalence of B2G-CIC (11.6%). This lower presence of B2G-CIC could be due to a more efficient IgG clearance-system. B2-CIC containing IgG or IgM have the capacity to activate complement, with the deposition of C3b and iC3b on these and making them accessible for the clearance in the liver and spleen by phagocytosis (through CR3 and CR4 receptors on macrophages) (83, 84). However, a lower clearance capacity of IgM-based B2-CIC through CR1 has been described compared to the removal of those formed by IgG (85). In addition, B2G-CIC can also be eliminated by the Fcγ receptors system present on immune cells (86). This additional mechanism is not present for B2-CIC formed by the IgM isotype. Only one receptor has been described for Fcμ of IgM and this receptor is involved in the regulation of immune response but does not perform clearance functions (87, 88). It may be possible that B2-CIC could only be detected in those patients when their clearance system is not sufficient enough to eliminate all of the B2-CIC because more quantity than normal has been generated.

The main limitation of our study is the absence of a control group made up of asymptomatic individuals with aPL (that is, individuals with aPL but with no history of APS clinical events and no concurrence of non-criteria symptoms associated with the syndrome). This group would be difficult to obtain because the evaluation of a large number of subjects would be necessary in order to recruit an adequate sample size of aPL-positive individuals without clinical manifestations and this would require extensive material and human investments. This is a retrospective study and in most of the cases the samples were not collected at the acute stage of the disease but rather during the follow-up. Since no longitudinal samples were included, it was not possible to determine whether the presence of B2-CIC increases at the time of the acute event and would thus constitute a risk factor for the development of APS events. In addition, it is difficult to find an association between the presence of B2-CIC and severe disease events due to the study design. All patients had a history of an APS event and 91% of them were taking preventive anticoagulant therapy, so the assessment of potential recurrences of severe clinical manifestations would be challenging. This work is fundamentally an epidemiological study that aims to find clinical and analytical associations with the presence of B2-CIC. For these reasons, further studies are needed to describe and confirm whether the observed thrombocytopenia and decreased C3 and C4 levels are caused by the binding of B2-CIC to the platelet surface and complement, respectively. Immunochemical studies should also be performed to confirm that immune-complexes integrating both B2GP1 and antibodies are capable of incorporating complement factors, which could initiate the activation of the classical pathway.

In conclusion, the presence of B2-CIC could contribute to higher complement and platelets consumption and therefore to a higher incidence of thrombocytopenia in patients with thrombotic APS.

## Data availability statement

The raw data supporting the conclusions of this article will be made available by the authors, without undue reservation.

## Ethics statement

The studies involving human participants were reviewed and approved by Clinical Research Ethics Committee of the Hospital Universitario 12 de Octubre, Madrid, Spain. The patients/participants provided their written informed consent to participate in this study.

## Author contributions

LN, LS, MS and AS conceived and designed the study. AD, LA, AT, MMaś, SS, MI, KK, MMan, FR, NS, MMil, JS, DR, IC, MR, MB, DP and YS contributed to the recruitment of patients, collection of samples and clinical data acquisition. LN and SG performed the B2-CIC quantification. LN, AS, and MS incorporated clinical and analytical information to the database, performed the first data analysis, wrote the first draft of the article and made all the changes suggested by the co-authors. All authors contributed to the article and approved the submitted version.

## Funding

This research was supported by "Instituto de Salud Carlos III (ISCIII), through the project PI20-01361 and co-funded by the European Union".

## Acknowledgments

We thank Margarita Sevilla and Carmen Caballero for their excellent technical assistance and Barbara Shapiro for the English revision and proof reading of this article.

## Conflict of interest

The authors declare that the research was conducted in the absence of any commercial or financial relationships that could be construed as a potential conflict of interest.

## Publisher’s note

All claims expressed in this article are solely those of the authors and do not necessarily represent those of their affiliated organizations, or those of the publisher, the editors and the reviewers. Any product that may be evaluated in this article, or claim that may be made by its manufacturer, is not guaranteed or endorsed by the publisher.
