# Supplementary material for: Circulating immune-complexes of IgG/IgM bound to B2-glycoprotein-I associated with complement consumption and thrombocytopenia in antiphospholipid syndrome
Source: Front Immunol. 2022 Sep 12;13:957201. doi: 10.3389/fimmu.2022.957201 (PMC9511106; doi:10.3389/fimmu.2022.957201)
Supplement: Supplementary file 1 [file DataSheet_1.docx]

Supplementary Material

**Supplementary Methods.** Antiphospholipid antibodies measurement (IgG/IgM aCL and aB2GP1 antibodies) by the six European centers participating in the study.

*Center 1: University Hospital Center Bezanijska Kosa (Belgrade, Serbia).* Antiphospholipid antibodies were measured by enzyme-linked immunosorbent assay (ELISA) (Binding Site Group Ltd, Birmingham, UK). They were expressed in G phospholipid (GPL) or M phospholipid (MPL) units (GPL-U and MPL-U, respectively).  The ranges for positive antibody levels were defined as low (11–40 U/ml), medium (41–99 U/ml) and high (>100 U/ml).

*Center 2: ASST Spedali Civili di Brescia* *(Italy).* The evaluation of aPL were performed by internationally validated in-house ELISA methods (1, 2). IgG/IgM aCL antibodies levels of 10-20 GPL/MPL were considered low positive, 20-80 GPL/MPL moderate positive and >80 GPL/MPL high positive. For aB2GP1 antibodies, 0.130-0.500 OD and 0.280-0.500 OD were considered low positives in the case of IgG and IgM, respectively. For both isotypes, 0.500-1.000 OD were considered moderate titers and >1.000 OD high titers.

*Center 3: National Institute of Geriatrics, Rheumatology and Rehabilitation (Warsaw, Poland).* The determination of aPL was performed also by an in-house ELISA. The cutoff values considered positive were >0.15 OD and 0.13 OD for IgG and IgM aB2GP1 antibodies, respectively. For IgG and IgM aCL, antibody levels >16 and >20 U/mL were considered positive, respectively.

*Center 4 and 5: San Giovanni di Dio Hospital (Florence, Italy) and San Giovanni Bosco Hospital (Torino, Italy).* IgG/IgM aCL and aB2GP1 antibodies were measured by a chemiluminescence immunoassay (BioFlash, Inova Diagnostics, San Diego, CA, USA). The cutoff values for IgG/IgM aCL/aB2GP1 antibodies were >20 CU/mL.

*Center 6: Hospital Universitario 12 de Octubre (Madrid, Spain).* IgG/IgM aCL and aB2GP1 antibodies were evaluated by ALBIA Multiplex Technology using BioPlex 2200 System (Bio-Rad Laboratories, CA, USA). Antibody levels higher than 18 U/mL were considered positive for IgG/IgM aCL/aB2GP1.

1. Tincani A, Allegri F, Balestrieri G, Reber G, Sanmarco M, Meroni P, et al. Minimal requirements for antiphospholipid antibodies ELISAs proposed by the European Forum on antiphospholipid antibodies. Thromb Res. 2004;114(5-6):553-8.

2. Pierangeli SS, Favaloro EJ, Lakos G, Meroni PL, Tincani A, Wong RC, et al. Standards and reference materials for the anticardiolipin and anti-beta2glycoprotein I assays: a report of recommendations from the APL Task Force at the 13th International Congress on Antiphospholipid Antibodies. Clin Chim Acta. 2012;413(1-2):358-60.

**Supplementary Table S1.** Number, age and sex of patients from the 6 European hospitals.

| **CENTER** | **No. PATIENTS** | **AGE (years)** | | **SEX (male/female)** | |
| --- | --- | --- | --- | --- | --- |
|  |  | **Median** | **IQR** | **N** | **Ratio** |
| Belgrade | 119 | 47.0 | 34.0-56.0 | 24/95 | 1:3.9 |
| Brescia | 38 | 39.5 | 34.0-52.0 | 10/28 | 1:2.8 |
| Florence | 15 | 58.5 | 54.0-70.0 | 7/8 | 1:1.1 |
| Madrid | 29 | 55.0 | 39.8-74.0 | 8/21 | 1:2.6 |
| Torino | 66 | 50.5 | 43.0-59.0 | 29/37 | 1:1.3 |
| Warsaw | 36 | 44.0 | 39.0-59.0 | 5/31 | 1:6.2 |
| **Total** | **303** | **47.0** | **38.0-57.0** | **83/220** | **1:2.7** |

**Supplementary Table S2.** Clinical and demographic characteristics of the multicenter cohort of 303 APS patients.

| **CONDITION** | **MULTICENTER COHORT OF APS PATIENTS**  (N=303) | |
| --- | --- | --- |
|  | **N / median** | **% / IQR** |
| Age (years) | 47.0 | 38.0-57.0 |
| Sex (women) | 220 | 72.6 |
| Disease duration (years) | 5.0 | 2.0-10.0 |
| Systemic lupus erythematosus | 112 | 37.0 |
| Primary APS | 165 | 54.5 |
| SAD-APS | 138 | 45.5 |
| Catastrophic APS | 8 | 2.6 |
| Dyslipidemia | 58 | 19.1 |
| Diabetes mellitus | 22 | 7.3 |
| Active smoker | 32 | 10.6 |
| Arterial hypertension | 62 | 20.5 |
| Antiplatelet agents | 206 | 68.0 |
| Anticoagulants | 269 | 88.8 |
| **APS PATHOLOGY** | | |
| Isolated thrombotic APS | 196 | 64.7 |
| Isolated gestational morbidity | 67 | 22.1 |
| Mixed APS | 40 | 13.2 |
| Total thrombotic APS | 236 | 77.9 |
| Total gestational morbidity | 107 | 35.3 |
| **Thrombotic events** |  |  |
| Arterial thrombosis | 124 | 40.9 |
| Venous thrombosis | 125 | 41.3 |
| Inferior extremity deep vein thrombosis | 93 | 30.7 |
| Superior extremity arterial thrombosis | 9 | 3.0 |
| Pulmonary embolism | 29 | 9.6 |
| Stroke | 64 | 21.1 |
| Acute myocardial infarction | 14 | 4.6 |
| **Gestational morbidity** |  |  |
| Early fetal loss | 93 | 30.7 |
| Late fetal loss | 35 | 11.6 |
| Mean fetal loss | 1.8 | 0.6 |

*Early fetal loss: miscarriage during the first 10 weeks, in the first trimester. Late fetal loss: miscarriage after the first 10 weeks of gestation.*

**Supplementary Table S3.** Positivity for B2-CIC when patients were differentiated according to their type of APS event.

| **TYPE OF EVENT** | **APS PATIENTS**  (N=303) | |
| --- | --- | --- |
|  | **Positive patients for B2-CIC (N)** | **%** |
| Isolated thrombotic APS (N=196) | 73 | 37.2 |
| Isolated gestational morbidity (N=67) | 35 | 52.2 |
| Mixed APS (N=40) | 11 | 27.5 |
| Total thrombotic APS (N=236) | 84 | 35.6 |
| Total gestational morbidity (N=107) | 46 | 43.0 |

*B2-CIC: presence of B2G and/or B2M-CIC*

**Supplementary Table S4.** Clinical, demographic and laboratory parameters of patients with thrombotic events (total thrombotic APS), positive and negative for B2-CIC (N=236).

| **PATIENTS WITH THROMBOTIC APS (N=236)** | | | | | | |
| --- | --- | --- | --- | --- | --- | --- |
| **CONDITION** | **B2-CIC positive**  **N=84, 35.6%** | | **B2-CIC negative**  **N=152, 64.4%** | | **p-value** | **OR / Hodges-Lehmann median difference (95% CI)** |
|  | **N/median** | **%/IQR** | **N/median** | **%/IQR** |  |  |
| Age (years) | 53.5 | 41.0-59.0 | 50.0 | 40.0-58.0 | 0.309 |  |
| Sex (women) | 57 | 67.9 | 96 | 63.2 | 0.470 |  |
| Disease duration (years) | 5.0 | 3.0-9.5 | 6.0 | 2.0-10.3 | 0.804 |  |
| Systemic lupus erythematosus | 35 | 41.7 | 61 | 40.1 | 0.819 |  |
| Primary APS | 42 | 50.0 | 82 | 53.9 | 0.562 |  |
| SAD-APS | 42 | 50.0 | 70 | 46.1 | 0.562 |  |
| Catastrophic APS | 1 | 1.2 | 7 | 4.6 | 0.265 |  |
| Early fetal loss | 14 | 16.7 | 29 | 19.1 | 0,647 |  |
| Late fetal loss | 6 | 7.1 | 17 | 11.2 | 0.317 |  |
| Arterial thrombosis | 39 | 46.4 | 84 | 55.3 | 0.194 |  |
| Venous thrombosis | 49 | 58.3 | 76 | 50.0 | 0.107 |  |
| Inf. extr. deep vein thrombosis | 35 | 41.7 | 58 | 38.2 | 0.396 |  |
| Superficial thrombophlebitis | 19 | 22.6 | 23 | 15.1 | 0.151 |  |
| Sup. extr. arterial thrombosis | 1 | 1.2 | 8 | 5.3 | 0.156 |  |
| Acute myocardial infarction | 6 | 7.1 | 8 | 5.3 | 0.559 |  |
| Stroke | 22 | 26.2 | 42 | 27.6 | 0.339 |  |
| Pulmonary embolism | 8 | 9.5 | 21 | 13.8 | 0.337 |  |
| Valve thickening, dysfunction | 5 | 6.0 | 1 | 0.7 | **0.015** | 9.60 (1.44-62.71) |
| Thrombocytopenia | 28 | 33.3 | 27 | 17.8 | **0.007** | 2.32 (1.25-4.28) |
| Hypocomplementemia | 26 | 31.0 | 33 | 21.7 | 0.140 |  |
| C3 levels (mg/dL) | 109.0 | 91.0-129.0 | 138.5 | 109.0-167.0 | **<0.001** | 29.0 (18.0-41.0) |
| C4 levels (mg/dL) | 18.9 | 13.0-26.8 | 27.7 | 18.6-37.4 | **<0.001** | 7.90 (4.20-11.88) |
| Platelets (x10^3^/μL) | 199.5 | 135.0-252.0 | 235.5 | 200.0-288.0 | **0.001** | 42.0 (16.0-68.0) |
| IgG aCL positive | 54 | 64.3 | 70 | 46.1 | **0.005** | 2.18 (1.26-3.79) |
| IgM aCL positive | 39 | 46.4 | 57 | 37.5 | 0.158 |  |
| IgG B2GP1 positive | 48 | 57.1 | 69 | 45.4 | 0.085 |  |
| IgM B2GP1 positive | 44 | 52.4 | 70 | 46.1 | 0.353 |  |
| LA positive | 66 | 78.6 | 112 | 73.7 | 0.405 |  |
| B2G-CIC positive | 29 | 34.5 | Na | Na | Na |  |
| B2M-CIC positive | 68 | 80.9 | Na | Na | Na |  |
| Triple aPL positivity | 48 | 57.1 | 64 | 42.1 | **0.027** | 1.83 (1.07-3.14) |
| Antiplatelet agents | 45 | 53.6 | 100 | 65.8 | 0.057 |  |
| Anticoagulants | 75 | 89.3 | 131 | 86.2 | 0.484 |  |
| Treated | 79 | 94.0 | 139 | 91.4 | 0.430 |  |

*aB2GP1: anti-β2-glicoproteína-I antibodies; aCL: anti-cardiolipin antibodies; LA: lupus anticoagulant; B2G-CIC: immune-complexes formed by B2GP1 and IgG aB2GP1 antibodies; B2M-CIC: immune-complexes formed by B2GP1 and IgM aB2GP1 antibodies.*

**Supplementary Table S5.** Comparison of variables previously identified with a p-value <0.050 between patients with isolated thrombotic APS, isolated gestational morbidity and mixed APS (independently of the presence of B2-CIC). For comparisons of qualitative variables with more than 2 categories, Pearson's X^2^ test was used for qualitative variables and Kruskal-Wallis’ test for quantitative variables.

| **CONDITION** | **THROMBOTIC APS**  **(N=196)** | **GESTATIONAL APS**  **(N=67)** | **MIXED APS**  **(N=40)** | **p-value** |
| --- | --- | --- | --- | --- |
|  | **N (%) /**  **median (IQR)** | **N (%) /**  **median (IQR)** | **N (%) /**  **median (IQR)** |  |
| Age (years) | 51.5 (41.0-59.0) | 39.0 (32.0-44.0) | 49.0 (37.5-56.5) | **<0.001** |
| Sex (women) | 113 (57.7%) | 67 (100%) | 40 (100%) | Na |
| Disease duration (years) | 5.0 (2.0-10.0) | 5.0 (1.8-10.0) | 6.0 (4.0-15.0) | 0.164 |
| B2G-CIC levels (U/mL) | 5.6 (3.1-10.5) | 3.8 (2.0-8.2) | 4.2 (2.2-6.0) | **0.006** |
| B2M-CIC levels (U/mL) | 11.0 (5.8-22.1) | 19.0 (8.2-30.8) | 10.1 (5.9-24.0) | **0.050** |
| B2G-CIC positive | 26 (13.3%) | 6 (9.0%) | 3 (7.5%) | 0.431 |
| B2M-CIC positive | 58 (29.6%) | 30 (44.8%) | 10 (25.0%) | **0.041** |
| B2-CIC positive | 73 (37.2%) | 35 (52.2%) | 11 (27.5%) | **0.025** |
| Platelets (x10^3^/μL) | 226.5 (176.0-281.0) | 230.0 (193.8-267.5) | 221.5 (184.0-248.5) | 0.607 |
| Thrombocytopenia | 42 (21.4 %) | 12 (17.9 %) | 13 (32.5 %) | 0.197 |
| C3 levels (mg/dL) | 124.0 (99.5-149.0) | 132.0 (106.0-160.0) | 147 (108.3-179.0) | 0.114 |
| C4 levels (mg/dL) | 23.0 (14.9-31.6) | 26.0 (18.4-36.3) | 30.2 (17.3-38.2) | 0.071 |
| Hypocomplementemia | 52 (26.5%) | 9 (13.4 %) | 7 (17.5 %) | 0.056 |
| IgG aCL positive | 104 (53.1 %) | 27 (40.3 %) | 20 (50.0%) | 0.218 |
| IgM aCL positive | 80 (40.8%) | 36 (53.7%) | 16 (40.0%) | 0.140 |
| IgG B2GP1 positive | 99 (50.5%) | 29 (43.3%) | 18 (45.0%) | 0.540 |
| IgM B2GP1 positive | 89 (45.4%) | 35 (52.2%) | 25 (62.5%) | 0.122 |
| LA positive | 145 (74.0%) | 42 (62.7%) | 33 (82.5%) | 0.066 |
| Triple positivity | 92 (46.9%) | 22 (32.8%) | 20 (50.0%) | 0.099 |
| Antiplatelet agents | 113 (57.7%) | 61 (91.0%) | 32 (80.0%) | **<0.001** |
| Anticoagulants | 170 (86.75%) | 63 (94.0%) | 36 (90.0%) | 0.180 |

*aB2GP1: anti-β2-glicoproteína-I antibodies; aCL: anti-cardiolipin antibodies; LA: lupus anticoagulant; B2G-CIC: immune-complexes formed by B2GP1 and IgG aB2GP1 antibodies; B2M-CIC: immune-complexes formed by B2GP1 and IgM aB2GP1 antibodies; B2-CIC: presence of B2G and/or B2M-CIC.*


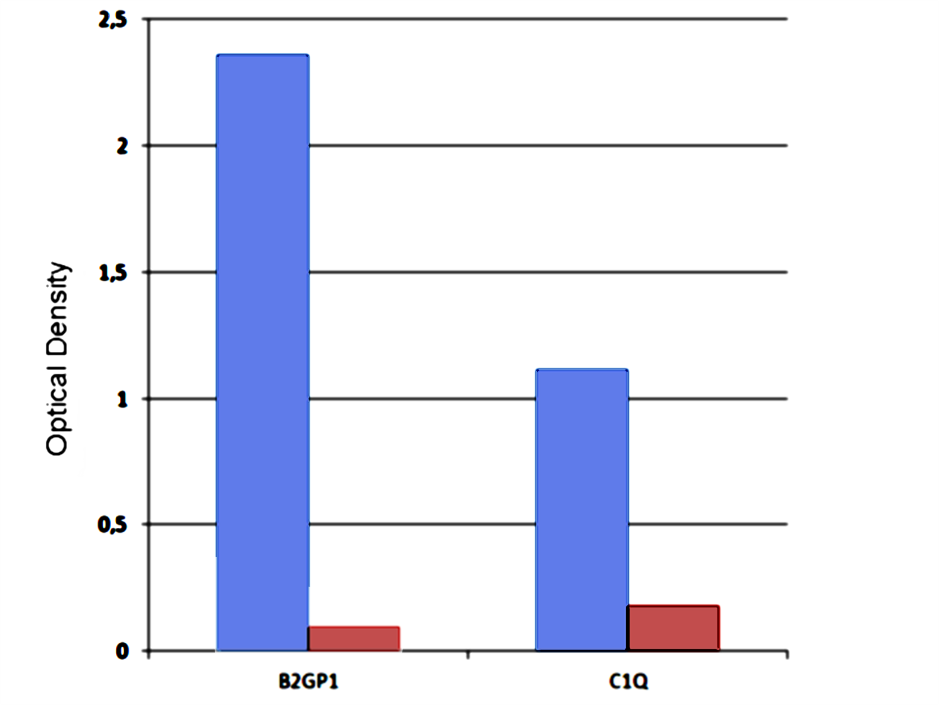


**Supplementary Figure S1.** Mean optical densities (OD) values of ELISA detection assays for human B2GP1 and human C1q in eluates of PEG-6000 precipitates from sera of six APS patients positive for immune-complexes (in blue) and two negative controls (in red).
